# Cilia protein IFT88 regulates extracellular protease activity by optimizing LRP-1–mediated endocytosis

**DOI:** 10.1096/fj.201800334

**Published:** 2018-06-19

**Authors:** Clarissa R. Coveney, Isabella Collins, Megan Mc Fie, Anastasios Chanalaris, Kazuhiro Yamamoto, Angus K. T. Wann

**Affiliations:** * Arthritis Research UK Centre for Osteoarthritis Pathogenesis, Kennedy Institute, Nuffield Department for Orthopaedics, Rheumatology, and Musculoskeletal Sciences, University of Oxford, Oxford, United Kingdom;; † Institute of Ageing and Chronic Disease, University of Liverpool, Liverpool, United Kingdom

**Keywords:** primary cilium, chondrocyte, cartilage, matrix, osteoarthritis

## Abstract

Matrix protease activity is fundamental to developmental tissue patterning and remains influential in adult homeostasis. In cartilage, the principal matrix proteoglycan is aggrecan, the protease-mediated catabolism of which defines arthritis; however, the pathophysiologic mechanisms that drive aberrant aggrecanolytic activity remain unclear. Human ciliopathies exhibit altered matrix, which has been proposed to be the result of dysregulated hedgehog signaling that is tuned within the primary cilium. Here, we report that disruption of intraflagellar transport protein 88 (IFT88), a core ciliary trafficking protein, increases chondrocyte aggrecanase activity *in vitro*. We find that the receptor for protease endocytosis in chondrocytes, LDL receptor–related protein 1 (LRP-1), is unevenly distributed over the cell membrane, often concentrated at the site of cilia assembly. Hypomorphic mutation of IFT88 disturbs this apparent hot spot for protease uptake, increases receptor shedding, and results in a reduced rate of protease clearance from the extracellular space. We propose that IFT88 and/or the cilium regulates the extracellular remodeling of matrix—independently of Hedgehog regulation—by enabling rapid LRP-1–mediated endocytosis of proteases, potentially by supporting the creation of a ciliary pocket. This result highlights new roles for the cilium’s machinery in matrix turnover and LRP-1 function, with potential relevance in a range of diseases.—Coveney, C. R., Collins, I., Mc Fie, M., Chanalaris, A., Yamamoto, K., Wann, A. K. T. Cilia protein IFT88 regulates extracellular protease activity by optimizing LRP-1–mediated endocytosis.

A range of cellular activities—beyond those that govern transcription, expression, and secretion—regulate tissue patterning and homeostasis. For example, receptor-mediated endocytosis exerts influence by removing signaling ligands (1) and enzymes (2, 3) from the extracellular space. Control of extracellular protease enzymes is critical in tissue remodeling, particularly in matrix-rich tissues, such as cartilage, where matrix anabolism and catabolism are carefully balanced by the only cell type present, the chondrocyte (4). Proteases have potent biochemical catabolic activity, which is kept strictly regulated in physiologic conditions. Musculoskeletal diseases, such as osteoarthritis (OA), are defined—and often diagnosed—by the catabolic destruction of cartilage tissue that lines the articular surfaces of joints without compensatory anabolic repair (5). Triggers and mechanisms of many forms of arthritis are still uncertain; however, there is broad agreement that increased protease activity drives pathologic cartilage matrix destruction (6, 7). Therefore, unveiling mechanisms for the physiologic regulation and pathologic dysregulation of protease activity remains critical to the understanding and treatment of arthritis and other diseases in which matrix turnover is altered.

In articular cartilage, extracellular matrix consists predominantly of collagen fibrils and proteoglycans, which form a tissue with the tensile strength and hydration that allows it to withstand constant mechanical challenge while also enabling smooth articulation. The activity of proteases, including the aggrecan proteoglycan-degrading aggrecanases, members of the a disintegrin and metalloproteinase with thrombospondin motifs (ADAMTS) family and collagenase members of the matrix metalloproteinase (MMP) family, are highly significant in arthritis (6, 7). *In vitro* studies, mouse models of OA, and late-stage clinical disease samples all implicate these enzymes in matrix destruction and pathogenesis; however, mRNA levels of the major aggrecanases of relevance—for example, *ADAMTS5*—do not always correlate to cartilage destruction. A number of upstream signaling pathways are implicated in their regulation, including NF-κB (8) and JNK (9). Recently, it has been demonstrated that extracellular protease levels—and most importantly, their activities—are regulated by cell uptake *via* the LDL receptor–related protein 1 (LRP-1) system (9–13). Reduced LRP-1–mediated uptake and shedding of the α (heavy chain) portion of LRP-1 is reported in OA chondrocytes and has been proposed to underlie the increased protease activity in the disease (12).

Chondrocytes, like most cell types, assemble a single primary cilium (14, 15), an organelle that became the subject of a number of reports after the discovery of the human ciliopathies at the turn of the 21st century (16). Congenital ciliopathies stem from mutations to proteins associated with the cilium, a collective termed the ciliome. The full ciliome is likely still unknown, but includes genes and proteins that are associated with ciliogenesis, ciliary structure, trafficking within the ciliary compartment, or receptors or signaling proteins that are enriched within the ciliary compartment. One of the first ciliary components defined in mammalian cells, intraflagellar transport protein 88 (IFT88), is a core anterograde or type B trafficking protein—carrying proteins to the tip of the primary cilium—and is therefore critical for ciliary assembly and function (17, 18). The most famous roles for the cilium, and certainly IFT, include the transduction of signaling downstream to Hedgehog (Hh) ligands (19), mechanical stimuli (20), and growth factors (21–23); however, the ciliome is now associated with the regulation of a plethora of cell biology through both ciliary and potentially nonciliary mechanisms. Microscopy studies of cilia in culture and *in situ*, including in tissues of the joint, such as synovium and cartilage, describe the association between the cilium, particularly the ciliary pocket, and vesicles of the endocytosis system (24–26). This is functionally important for cilia, downstream to growth factors and Hh (22, 27); however, it remains unknown whether the ciliary pocket is as important to global cellular endocytosis in mammalian cells as the analogous flagellar pocket is for trypanosomes (28).

Many ciliopathies have striking musculoskeletal phenotypes (29). When core ciliome components, including IFT88, have been deleted during development in murine models, skeletal morphology and organization is grossly disrupted, including at the level of matrix deposition and thus potentially turnover (30–34). Whereas Hh signaling is activated in OA (35), the activation of the pathway alone does not drive cartilage catabolism (36). In chondrocytes, ciliary IFT is also important for the cellular response to pathophysiologically relevant stimuli, such as mechanics, inflammatory cytokines, and osmotic flux (25, 37–40). Here, we experimentally examined whether the ciliome, specifically IFT88, directly regulates matrix catabolism independent of transducing pathophysiologic stimuli.

## MATERIALS AND METHODS

### Primary Abs, substrates, recombinant proteins, and inhibitors

The following primary Abs were used in this study: Aggrecan (6-B-4) (Abcam, Cambridge, MA, USA), ARGXX (BC-3) (Abcam), phospho-JNK1/2 (Thermo Fisher Scientific, Waltham, MA, USA) β-actin (Cell Signaling Technology, Danvers, MA, USA), FLAG-M2 (MilliporeSigma, Burlington, MA, USA), acetylated α-tubulin (6-11B-1) (MilliporeSigma), early endosome antigen 1 (EEA-1; Abcam), LRP-1 β-light chain (Abcam), LRP-1 α-heavy chain (8G1) (Abcam), a disintegrin and metalloproteinase (ADAM)-17 (Abcam), MMP-13 (Santa Cruz Biotechnology, Dallas, TX, USA), MMP14 (anti-catalytic domain EP1264Y) (Abcam), mouse monoclonal anti–MT1-MMP hemopexin domain 222-1D8 (a gift from Yoshi Itoh, Kennedy Institute) generated as previously described (41), and arl13b (ProteinTech, Rosemont, IL, USA). Anti-AGEG was a kind gift from Hideaki Nagase (University of Oxford). Purified aggrecan from bovine cartilage came from MilliporeSigma. Recombinant human C-terminal His-tagged human receptor-associated protein (RAP) was produced in *Escherichia coli* using a pET3a-based expression vector and purified as described previously (13). The domain deletion mutant, ADAMTS-5 (TS5-3)-flag, is described in Gendron *et al*. (42), and recombinant MMP-13 is described in Yamamoto *et al*. (43). Recombinant IL-1β was purchased from PeproTech (London, United Kingdom).

### Cell culture

The majority of experiments used a chondrocyte cell line as described in Results and previously (37). The line was maintained and immortalized at 33°C in low-glucose DMEM that was supplemented with 10% (v/v) fetal calf serum, 2.5 mM l-glutamine, 88 U/ml penicillin, 90 µg/ml streptomycin, and 10 ng/ml IFN-γ. Experiments were conducted using primary cells by removing IFN-γ and culturing at 37°C. For experiments that used primary human chondrocytes, cartilage samples were obtained from the Oxford Musculoskeletal Biobank, collected with informed donor consent before knee replacement surgery, in full compliance with national and institutional ethical requirements, the United Kingdom Human Tissue Act, and the Declaration of Helsinki (HTA License 12217 and Oxford REC C 09/H0606/11). Cartilage explants were removed from the joints immediately after surgery, diced, and subjected to collagenase digestion (type A; Roche, Basel, Switzerland) at 2 mg/ml overnight at 37°C in primary medium [low-glucose DMEM that was supplemented with 10% (v/v) fetal calf serum, 2.5 mM l-glutamine, 88 U/ml penicillin, 90 µg/ml streptomycin, and 16 mM HEPES]. Tissue suspension was then filtered through a 70-µm filter. Cells were then resuspended in primary medium, counted, and seeded onto 13-mm glass coverslips at 2 × 10^4^/cm, then cultured for up to 48 h or to confluence to ensure quiescence.

### Aggrecanase assays

We used a system of coculture with aggrecan as described previously (9). Chondrocytes were cultured in serum-free medium that contained 50 µg/ml bovine aggrecan using IL-1β (50 ng/ml) as a positive control, and medium was collected at time points after this in a 24-h period. Before analysis, 150 μl of medium sample was deglycosylated overnight at 37°C using Chondroitinase ABC (0.0013 U/mg GAG; MilliporeSigma) and endo-β-galactosidase from *Bacteroides fragilis* (0.0007 U/mg GAG; MilliporeSigma) added in 50 μl of digestion buffer that consisted of Tris (50 mM), EDTA (25 mM), and 50 mM sodium acetate (pH 7.5). Proteins were precipitated with 1 ml of ice-cold acetone and the pellet was air dried before resuspension in 50 μl Laemmli loading buffer (Bio-Rad, Hercules, CA, USA) that contained 2-ME (1:10) for loading. Fragments that were generated by cleaving at the Glu^1819^-Ala^1820^ bond in aggrecan were detected using a polyclonal rabbit Ab directed at N-terminal AGEG. Samples were run on 4–12% NuPAGE Bis-Tris gels, then transferred to PVDF membrane. Bands were detected using ECL Plus Reagent, imaged on a Syngene G:Box imager (Fredrick, MD, USA).

### Quantitative RT-PCR

Cells were cultured per aggrecanase experiments, then RNA was isolated using an RNAeasy Mini Kit (Qiagen, Germantown, MD, USA). RNA was resuspended in RNAase-free water and yields and indicative purity were checked using Nanodrop (Thermo Fisher Scientific). One microgram of RNA was used to synthesize cDNA, and reverse transcription was performed using the ABI High Capacity Kit (Thermo Fisher Scientific) following the manufacturer’s instructions. Real-time RT-PCR was performed using the TaqMan (FAM dye) Universal Master Mix II (Thermo Fisher Scientific). Each reaction consisted of 0.5 µl cDNA template, 5 µl Master Mix, 0.5 µl primer, and 4 µl nuclease-free water. Samples were loaded in a 384-well plate and thermocycling was performed on a ViiA7 Real-Time RT-PCR System (Thermo Fisher Scientific) using the following protocol: hold 2 min at 50°C; hold 10 min at 95°C; 40 cycles: 15 s at 95°C, 1 min at 60°C; hold at 4°C. Data were captured and primary analysis was performed using Expression Suite Software (v.1.1; Applied Biosystems, Foster City, CA, USA) using the ΔΔ*C_t_* method using 18S as a normalizing gene. TaqMan assays (**Table 1**) were purchased from Thermo Fisher Scientific.

**TABLE 1:** TaqMan probes used for quantitative RT-PCR

Gene	Catalog reference	Amplicon length (bp)	Reference sequence
*RPS18*	Mm02601776_m1	74	NM_011296.2
*ADAMTS1*	Mm00477355_m1	98	NM_009621.4
*ADAMTS4*	Mm00556068_m1	66	NM_172845.2
*ADAMTS5*	Mm00478620_m1	103	NM_011782.2
*MMP3*	Mm00440295_m1	66	NM_010809.1
*MMP13*	Mm00439491_m1	65	NM_008607.2
*TIMP3*	Mm00441826_m1	59	NM_011595.2
*GLI2*	Mm01293116_m1	80	NM_001081125.1
*GLI3*	Mm00492337_m1	82	NM_008130.2
*PTCH1*	Mm00436026_m1	69	NM_008957.2

### Western blotting

#### Cell lysates

Cells were kept on ice and washed quickly in ice-cold PBS before ice-cold lysis buffer (150 mM sodium chloride, 1% Triton X-100, 50 mM Tris, pH 8, a cocktail of protease inhibitors; Roche) was added to cells for 5 min. Cells were removed from the culture surface using a cell scraper before being left for an additional 5 min and homogenized using a 21-gauge needle. Samples were spun at 10,000 rpm for 15 min at 4°C before the supernatant cytoplasmic fraction was frozen and stored at −20°C.

#### Conditioned medium

Cells were cultured as per before aggrecanase experiments, then serum-free medium was collected after 24 h and stored at −20°C. Proteins were resolved by Bis-Tris gels, with the exception of LRP-1α for which samples were run, undenatured, on TCA gels. For endocytosis assays and LRP-1β expression, near-infrared secondary Abs (Li-Cor, Cambridge, United Kingdom) were used, multiplexing target protein with a reference where possible, and ImageJ (National Institutes of Health, Bethesda, MD, USA) was used to quantify immunoreactive bands. Other blots—neoepitopes, shed LRP-1, and sheddase expression—used horseradish peroxidase secondary Abs and ECL Plus detection.

### Endocytosis uptake assays

Cells were cultured as previously described. After 72 h, cells were washed with PBS and cultured in serum-free medium for 24 h before medium was replaced, again with serum-free medium, for the duration of uptake experiments. Cells were cultured with serum-free medium with a final concentration of 20 nM FLAG-tagged ADAMTS-5 or 10 nM Pro-MMP-13. Where the experimental design required, cells were coincubated with a final concentration of 500 nM RAP for 20 min before the addition of ligands. Medium samples were collected straight into Laemmli loading buffer (Bio-Rad), boiled for 5 min, and frozen.

### Immunofluorescence

Cells were cultured to confluence on 1.9-cm^2^ glass coverslips. For total cell staining, coverslip cultures were fixed using 4% paraformaldehyde at 37°C for 7 min, followed by permeabilization with 0.5% Triton X-100, and were blocked with 5% goat serum. Coverslips were incubated overnight at 4°C with primary Abs, depending on the protein of interest: acetylated α-tubulin (1:2000), arl13b (1:1000), LRP-1 (1:1000), FLAG-TS-5 (1:500), and EEA-1 (1:500). Coverslips were washed and incubated for 45 min in the dark at 25°C with Alexa Fluor 488 anti-mouse conjugate (Thermo Fisher Scientific) and Alexa Fluor 555 anti-rabbit conjugate (Thermo Fisher Scientific) before mounting with DAPI (1:5000) counterstain (Thermo Fisher Scientific). For surface staining, coverslip cultures were only fixed for 1 min and no permeabilization was conducted before blocking with 5% goat serum. Samples were then treated the same as previously described. Coverslips were mounted onto slides using Prolong Gold (Thermo Fisher Scientific and sealed. For FLAG-TS-5 uptake/cilia length studies, wild-type cells were treated with FLAG-TS-5 over a 60-min time course before fixation as per total cell protocols. Images shown are from 40-min time point.

Imaging and analysis images were acquired using an Olympus FluoView FV1000 Confocal Microscope (Olympus, Tokyo, Japan) with an oil immersion ×63 objective to produce confocal serial sections for maximum-intensity *z*-stack reconstruction of monolayer fields with laser voltage, offset and gain held constant. Cilia length was measured using a medial line that was defined along the center of the cilium using ImageJ software. Analysis of LRP-1β signal distribution was conducted using two methods. The first used solely the LRP-1β signal for each field and an ImageJ histogram analysis was run before plotting the pixel intensity data in Prism 6 (GraphPad Software, La Jolla, CA, USA). A second manual approach classified single-cell LRP-1β signal into one of 4 groups: *1*) singular focal staining, *2*) majority of staining in 1 quadrant, *3*) in 1 hemisphere, or *4*) evenly distributed. Quadrants were assigned, square to edge of image and centered on nuclei. These were subsequently analyzed as contingency data using a χ^2^ test.

### Data presentation and statistics

Graphs were generated in Prism 6. All data analyses were performed in Prism. Parametric and nonparametric tests were chosen after D’Agostino-Pearson omnibus normality tests. Statistical tests were used as described in Results.

## RESULTS

### Hypomorphic mutation to IFT88 results in constitutive aggrecanase activity

Our first aim was to assess whether protease activity changed upon experimental disruption of the ciliome *in vitro*. The IFT88 Oak Ridge Polycystic Kidney (IFT88*^ORPK^*) mutation is hypomorphic, which results in a shortened form of the protein, but is not 100% penetrant in terms of cilia phenotype (15% of chondrocytes *in vitro* assemble stunted cilia) (33, 37). Using a previously described coculture system (9), we added bovine aggrecan to wild-type and IFT88*^ORPK^* mouse chondrocyte cultures and probed the culture medium for aggrecan and aggrecanase neoepitopes.

**Figure 1:**
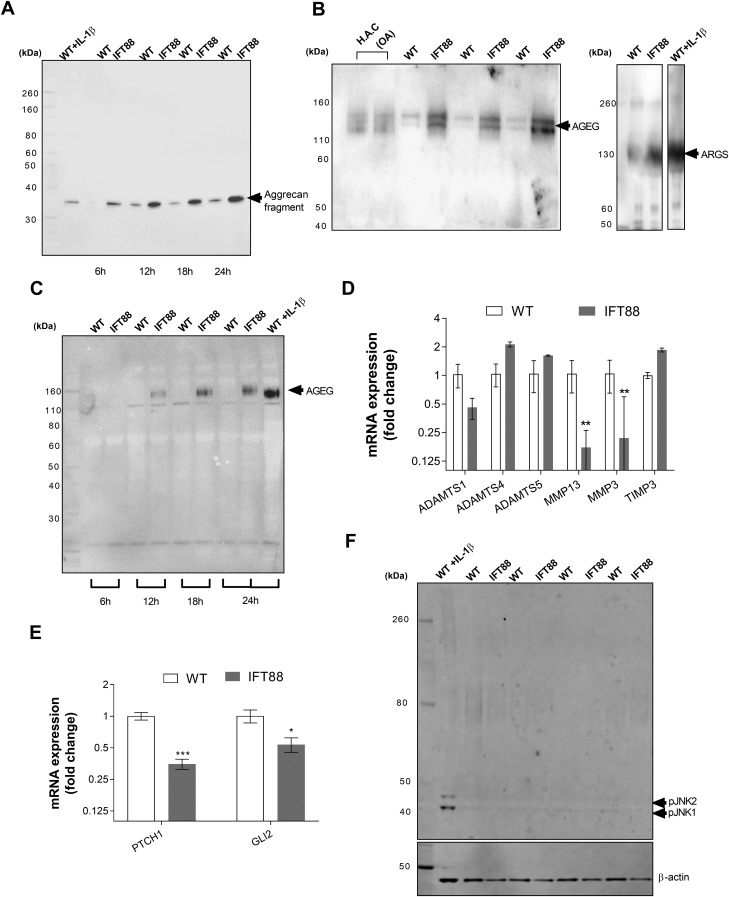
IFT88 mutation results in constitutive catabolic activity without associated transcriptional regulation. Wild-type (WT) and IFT88*^ORPK^* chondrocytes were cultured with full-length aggrecan medium collected at intervals. *A*) Western blot analysis of conditioned medium, probing for aggrecan showing 35-kDa fragment in cultures that were treated with 10 ng/ml IL-1β and over time in unstimulated cultures. *B*, *C*) Western blot analysis showing the presence of AGEG and ARGS neoepitopes in conditioned medium after 24 h (*B*), and AGEG over a time course (*C*). *D*, *E*) Quantitative RT-PCR analysis of WT and IFT88 chondrocytes from identical experiments. Data shown are mean Δ*C_t_* normalized to average WT ± sd. ***P = 0.0004, *P = 0.008 for PTCH1 and GLI2, respectively, multiple Student’s *t* test corrected by Holm-Sidak method; *n* = 3). *F*) Western blot analysis of cell lysates probed for pJNK1 and pJNK2, with positive control WT cells treated with 10 ng/ml IL-1β and lysate collected at 10 min (*n* = 4).

Treatment with IL-1β for 24 h resulted in the appearance of a 35-kDa aggrecan fragment, which was indicative of proteolysis of full-length aggrecan (∼250 kDa, core protein). An identical band appeared without cytokine stimulation after 12 h in wild-type cultures but earlier, at 6 h, in IFT88*^ORPK^* cultures that remained more pronounced in IFT88*^ORPK^* at all time points (**Fig. 1*A***). IFT88*^ORPK^* chondrocytes also had increased production of aggrecan neoepitopes, AGEG and ARGS (Fig. 1*B*), at 24 h. Neoepitope bands were comparable in size to those produced by primary human articular chondrocytes that were isolated from osteoarthritic joints and wild-type mouse chondrocytes that were treated with IL-1β for 24 h (Fig. 1*B*). Relative aggrecanase activity, assessed by probing for AGEG over 24 h, was consistently higher in IFT88*^ORPK^* cultures, apparent first at 12 h when there was little measurable activity in WT cultures (Fig. 1*C*). To explore the underlying mechanism for this enhanced aggrecanase activity, we first investigated the gene expression profiles of a number of cartilage proteases using quantitative RT-PCR. We found no transcriptional signature to explain this increased activity. There were no increases in *ADAMTS1*, -*4* or, -*5*; *MMP3* or -*13*; or indeed a difference in mRNA levels of the tissue inhibitor of metalloproteases (*TIMP3*; Fig. 1*D*). RT-PCR analysis also demonstrated that, in these experimental conditions, IFT88*^ORPK^* mutation results in the down-regulation of the Hh pathway, previously proposed to drive cartilage destruction in OA (35), as indicated by reduced expression of pathway target patched-1 (*PTCH1*) and transcription factor glioma-associated oncogene family zinc finger 2 (*GLI2*) (*P* = 0.0004 and 0.008, respectively, multiple Student’s *t* test corrected using Holm-Sidak method; *n* = 3; Fig. 1*E*). We have previously reported that this mutation influences the response of chondrocytes to cytokines by regulating NF-κB signaling, itself a proposed regulator of aggrecanase transcription (8); however, as reported (38), there is no constitutive pathway activation. Similarly, here we found no constitutive JNK pathway activity (Fig. 1*F*), also previously linked to aggrecanase activity (9). These data implied that IFT88 regulates aggrecanase activity by post-transcriptional means.

### Polarized endocytosis of ADAMTS-5 at the primary cilium

**Figure 2:**
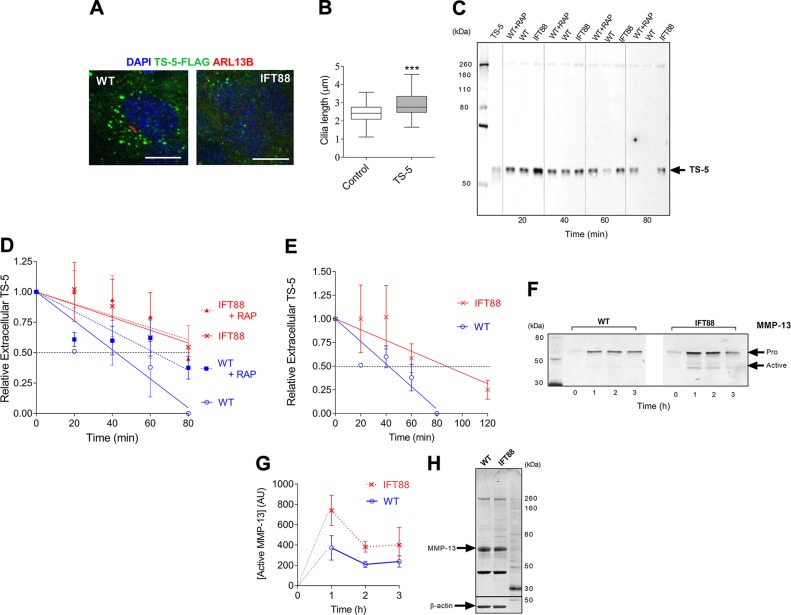
Perturbation of IFT88 impairs chondrocyte endocytosis of proteases. Cells were cultured with recombinant proteases—either FLAG-TS-5 or full-length MMP-13—and uptake of proteases was monitored by microscopy or measurement of FLAG-TS-5 in extracellular media by Western blot. *A*) Confocal fluorescent microscopy of wild-type (WT) and IFT88*^ORPK^* cells on exposure to FLAG-TS-5 (green). Primary cilia (red) were labeled with anti-arl13b and nuclei with DAPI (blue). Scale bar, 10 μm. Image captured after 40 min of incubation. *B*) Cilia length data as shown by box plot with Tukey whiskers. ***P ≤ 0.001 (Mann-Whitney *U* test, control *n* = 61 cilia; FLAG-TS-5, *n* = 62 cilia). Images also taken at the 40-min incubation time point. *C*, *D*) Western blot analysis of extracellular FLAG-TS-5 over time(*C*) and quantification of data (data are shown as mean normalized to 0 time-point, ± sd, *n* = 6; *D*). *E*) Quantification of repeat study comparing WT and IFT88*^ORPK^* cells over an extended time course (means ± sd, *n* = 3). *F*, *G*) Western blot for rMMP-13 uptake showing both full-length (pro) and cleaved (active) MMP-13 (*F*) and quantification of data (means ± sd, *n* = 3; *G*). *H*) Western blot for MMP13 expression in cell lysates.

Recent research has shown that chondrocyte aggrecanase activity is regulated post-transcription by endocytotic clearance *via* the LRP-1 system (13), disturbed in OA (44). The primary cilium has been described as a site that is enriched for endocytotic activity *in situ* (25). To investigate the possibility that the disruption of IFT88 and the primary cilium might affect LRP-1–mediated endocytosis of aggrecanases, we cocultured cells with FLAG-tagged, ADAMTS-5 (TS5-3) (42), termed FLAG-TS-5, and monitored uptake. We first cultured chondrocytes on glass coverslips to observe uptake by microscopy. Cell-associated signal was visible from 30 min after the addition of ligand to cultures. At 40 min, wild-type chondrocytes exhibited greater levels of cell-associated FLAG-TS-5 than IFT88*^OPRK^* chondrocytes (**Fig. 2*A***, green signal). We also observed that, in wild-type cells, this signal was preferentially localized to one side of the cell, at its center, the site of ciliary assembly (red, anti-arl13b signal Fig. 2*A*, expanded image). While imaging aggrecanase uptake, we observed an increase in cilia length (15% increase in median length, Fig. 2*B*). This implied an interplay between ciliary IFT and the uptake of ADAMTS-5.

### Endocytosis of protease impaired by IFT88 mutation

To explore in a more quantitative manner the possibility that IFT88 regulates the rate of endocytotic clearance of proteases, we measured extracellular (culture medium) levels of recombinant FLAG-TS-5 over time. After its addition to cells, levels of extracellular FLAG-TS-5 rapidly dropped, with signal often absent by 80 min (Fig. 2*C*). Quantification of repeat experiments (Fig. 2*D*) demonstrated that this uptake was partially blocked by the LRP-1 antagonist, RAP, and that extracellular FLAG-TS-5 levels were consistently higher in IFT88*^ORPK^* cultures. The extracellular half-life of FLAG-TS-5 in IFT88*^ORPK^* cultures was double that of wild-type cultures, which was indicative of a reduced rate of LRP-1–mediated endocytotic uptake. The extracellular half-life was similar or greater to that observed in wild-type cells inhibited by RAP. Rates of uptake in IFT88*^ORPK^* cultures were unaffected by the addition of RAP. An extended time course revealed that there was, on average, still 25% of FLAG-TS-5 remaining at 2 h after addition to IFT88*^ORPK^* cells, 40 min after the signal became unmeasurable in wild-type cells (Fig. 2*E*). Repeat studies using recombinant MMP-13, also an LRP-1 ligand, demonstrated a reduced rate of uptake for MMP-13 in IFT88*^ORPK^* cultures (Fig. 2*F*), associated with the accumulation of cleaved, active MMP-13 (quantified in Fig. 2*G*). Importantly, both cell types secreted low amounts of endogenous MMP-13 protein into the culture medium. Furthermore, in contrast to mRNA levels, protein expression was unchanged in IFT88*^ORPK^* lysates (Fig. 2*H*).

### Disruption of IFT88 stimulates LRP-1 shedding

**Figure 3:**
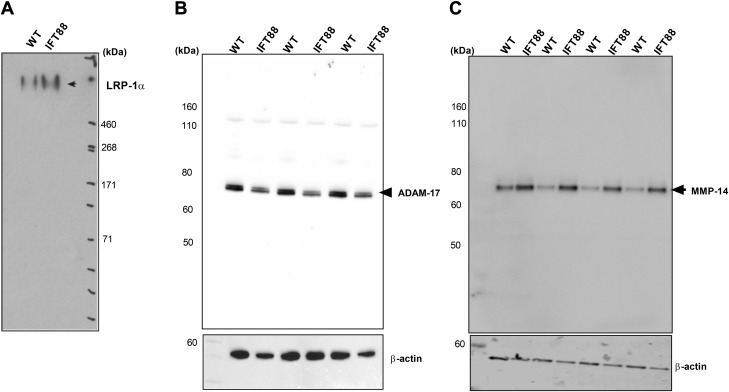
IFT88 mutation results in LRP-1 shedding. *A*) Western blot for extracellular (shed) LRP-1α in conditioned medium of cultures. *B, C*) Western blot of cell lysates that demonstrates the cellular expression of sheddases ADAM-17 (*n* = 3; *B*) and MMP-14 (*n* = 4; *C*), respectively. Conditioned medium and lysates for all groups were collected after 24 h of culture.

The light chain (β) of LRP-1 is cleaved by sheddases at the cell surface, and the entire heavy chain (α) is released into the extracellular milieu, a process that is enhanced in OA and that leads not only to reduced uptake of ligands by endocytosis, but also to enhanced extracellular activity of proteases by binding to the protease (12). We hypothesized that this LRP-1 may have been shed and present at higher levels with disruption of IFT. By probing conditioned media, we found that extracellular amounts of high-MW LRP-1α subunit were higher in IFT88*^ORPK^* cultures (**Fig. 3*A***), which indicated that the mutation is associated with enhanced shedding of LRP-1. Previous studies identified 2 sheddases of LRP-1 in chondrocytes, ADAM-17 and MMP-14 (12). Kidney epithelial cells with the identical IFT88*^ORPK^* mutation have previously been described to have elevated ADAM-17 (45), but, in contrast, we found that IFT88*^ORPK^* chondrocytes expressed similar or lower levels of ADAM-17 than wild-type chondrocytes (Fig. 3*B*); however, we found increased cellular expression of MMP-14 in IFT88*^ORPK^* chondrocytes compared with wild-type cells (Fig. 3*C*).

### Spatial association between microtubule organizing center/primary cilium and LRP-1

**Figure 4:**
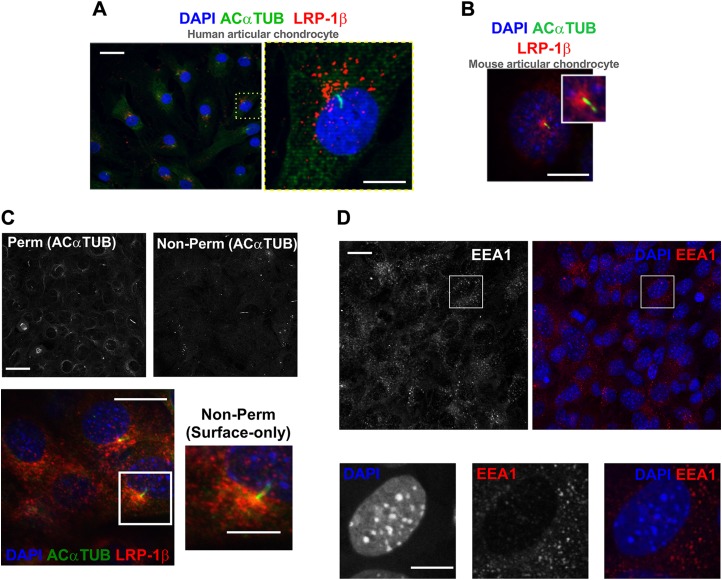
Membranous hot spot of LRP-1β associated with primary cilium. *A*) Immunofluorescent staining of primary cilia (green) and LRP-1β (red) in human articular chondrocytes. *B*) Immunofluorescent staining of LRP-1β (red) and primary cilia (green) in wild-type (WT) mouse chondrocytes. *C*) Immunofluorescence images shows surface-only staining of LRP-1β (red) in WT cells that concentrated at the base of the primary cilium (green) in cells without permeabilization as validated by the absence of a majority of acetylated α-tubulin signal (black and white images above). *D*) Immunofluorescence of EEA-1 (red) in mouse WT chondrocytes. Nuclei were labeled with DAPI. Scale bars, 10 µm.

**Figure 5:**
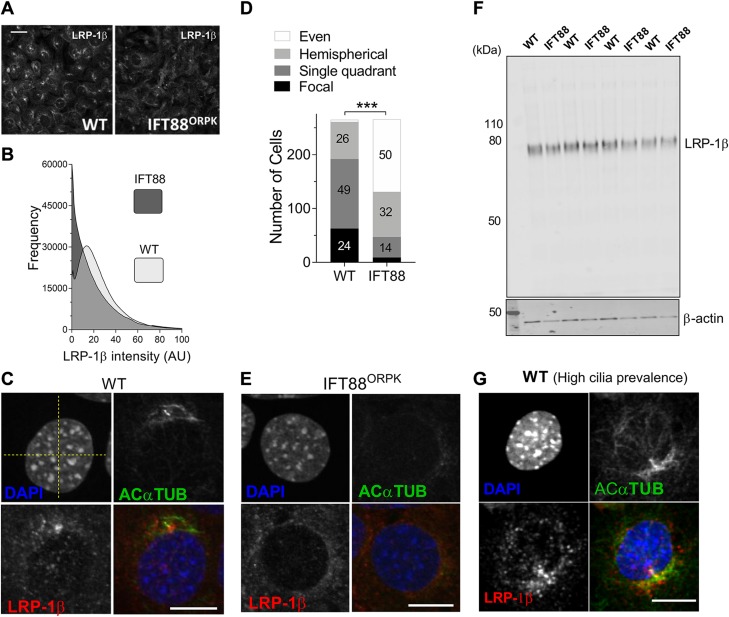
IFT88 mutation disturbs hot spot that is possibly associated with the ciliary pocket. *A*) Fluorescent microscopy of wild-type (WT) and IFT88*^ORPK^* cultures demonstrates the LRP-1β signal distribution in field of cells. *B*) Frequency histogram of LRP-1β signal intensity distribution for 6 fields of cells (~*n* = 300 cells). *C, E*) Single-cell confocal images of WT (focal distribution; *C*) and IFT88*^ORPK^* (even distribution; *E*) cells. Yellow dashed lines were used to define quadrants centered on nuclei. *D*) Contingency data from a classification analysis from 7 fields of WT and IFT88*^ORPK^* cultures that describe numbers in each group (percentages shown on bars, with the exception of WT even distribution, which had 1% frequency, and IFT88*^ORPK^* single focal distribution, which had 4% frequency) of each type of staining within total populations. Statistical difference is from a χ^2^ analysis. ***P < 0.0001. *F*) Western blot analysis of LRP-1β total protein expression (*n* = 4 repeats). *G*) LRP-1β (red) signal localized along length of the cilium (green), potentially lining a ciliary pocket (representative of single quadrant distribution for classification data). Scale bars, 10 μm.

In light of the observation that FLAG-TS-5 accumulated in a polarized fashion in chondrocytes (Fig. 2*A*), and previous reports of endocytosis at the base of the primary cilium (24, 25), we next explored LRP-1 distribution by microscopy. Both human primary and murine chondrocytes were stained with an Ab to the light (β) chain of LRP-1 (red), which includes the transmembrane and short cytoplasmic domains. We also colabeled primary cilia; staining for acetylated α-tubulin (green) and nuclei are blue throughout (DAPI). In both human primary chondrocytes (**Fig. 4*A***) and mouse wild-type chondrocytes, (**Figs**. 4*B* and **5*A***, wide-field), LRP-1β was predominantly expressed in a perinuclear manner and associated with the microtubule network, but often formed into bright clusters and concentrations near the microtubule organizing center (MTOC), the site of cilia assembly. LRP-1β expression, on occasion, was nearly exclusively confined to a singular signal at the ciliary base, but more commonly observed to be highly polarized to this area (as described and quantified in Fig. 5). Preparation of samples—without a permeabilization step and with only a brief paraformaldehyde fixation—resulted in staining bias toward near cell surface only. This was validated by the observation that cytoplasmic signal—indicated by acetylated α-tubulin—becomes barely visible apart from cytokinetic bridges (Fig. 4*C*, black and white images). In these conditions, LRP-1β distribution displayed the same polarized distribution, which implied the periciliary organization of LRP-1 on the membrane (Fig. 4*C*, red signal). In contrast, cytoplasmic early EEA-1, which is reflective of internalized early endosomes, was not similarly polarized (Fig. 4*D*, red signal).

### IFT88*^ORPK^* mutation disrupts LRP-1 organization

In IFT88*^ORPK^* chondrocytes, LRP-1β expression was more homogenously distributed over the cells compared with wild-type cells, although some polarization of signal is still observed in one quadrant of the cell and sometimes at the site of MTOC (Fig. 5*A, C, E*). This staining was imaged in multiple fields from multiple cell preparations (*n* ≥7 fields, >300 cells), all of which demonstrated qualitative changes to the surface distribution of LRP-1β. ImageJ analysis of the frequency distribution of LRP-1β signal in these whole-field images indicated a change in the frequency of distribution of signal intensity between wild-type and IFT88*^ORPK^* populations (Fig. 5*B*). Additional classification of fields of cells into 4 subtypes of LRP-1β distribution revealed a statistically significant difference between wild-type and IFT88*^ORPK^* cells (*P* ≤ 0.0001, χ^2^ test; *n* = 7 fields, at least 265 cells/population). Approximately 75% of wild-type cells had the majority of the LRP-1β signal in one quadrant of the cell or a single focal point at the site of ciliary assembly. In comparison, >80% of IFT88*^ORPK^* cells had even distribution or a hemispherical polarization (Fig. 5*D*). Total LRP-1β protein expression, as measured by Western blot, was not different (Fig. 5*F*). In cell preparations, ciliation in wild-type cells can range from 50 to 80%, increasing with cell quiescence. In adult mouse cartilage tissue, cilia prevalence is usually high [>50% and polarized in position (33)]. In culture, when ciliation was greatest (70–80% prevalence) a ciliary axoneme-associated signal for LRP-1β was commonly observed (Fig. 5*G*).

To summarize, we find that hypomorphic mutation to IFT88 in chondrocytes results in marked increases in aggrecanase activity, seemingly independent of transcriptional regulation. This is associated with reduced endocytotic clearance of protease, potentially as a critical organization of membrane LRP-1 is disrupted. Disruption of this organization of membranous LRP-1β is also associated with the shedding of LRP-1α, which likely contributes to the endocytotic and aggrecanase phenotypes. We propose that the polarized, periciliary region is important for efficient, rapid endocytosis of protease and is important to the regulation of matrix remodeling.

## DISCUSSION

Previous studies have linked the primary cilium and/or its associated core protein machinery with musculoskeletal development (46), matrix patterning (33), and turnover processes (32), as well as, potentially, the development of OA *via* ciliary-regulated Hh signaling (32, 35). A key component of the cilium’s machinery, IFT88 is highly influential in cartilage (33) and transduces the chondrocyte response to mechanical (37) and inflammatory cues (39). Although canonical, ciliary Hh signaling is hugely influential, activation of Hh alone does not activate cartilage catabolism *in vitro* (36). Here, we directly explored the role of the cilium in the regulation of protease activity. Hypomorphic disruption of IFT88 increased the constitutive catabolic activity of chondrocytes *in vitro*. The IFT88*^ORPK^* mutation, which results in changes to matrix patterning in cartilage *in vivo* (33), renders cells unable to express full-length IFT88 and assemble a primary cilium. This has also previously been associated with the regulation of multiple chondrocyte responses (37–39, 47–49), most of which are proposed to be indicative of roles for the cilium. Importantly, however, IFT88, and indeed this mutation, are both associated with cell biology that is not proven to be attributable to cilia function—for example, migration (50), cell division (51), and planar cell polarity (52).

Weak inherent gelatinase activity and collagenase activity in these cells make it difficult to assess these protease activities by zymography and collagen II coculture, respectively; however, using a recently developed technique (9) that allows for the assessment of aggrecan degradation by quantifying the production of aggrecan neoepitopes, we observed increased constitutive aggrecanase activity stimulated in IFT88*^ORPK^* chondrocytes. This activity was comparable to that of human primary chondrocytes from the clinical OA setting or mouse chondrocytes that are stimulated with the inflammatory cytokine, IL-1β. Relative rates of AGEG production between wild-type and IFT88*^ORPK^* cells seemed to diverge over a 24-h time course, which implies an immediate inherent difference in the cells, which we would now propose to be impaired receptor-mediated endocytosis of aggrecanase enzymes, and a synergistic factor, which we propose to be LRP-1 shedding. In OA, tissue damage is not always directly associated with increased gene expression of catabolic enzymes. In our experimental setting, ADAMTS expression was unchanged, whereas MMP expression was reduced. A number of candidate pathways lie upstream in the regulation of aggrecanase activity. The NF-κB pathway is upstream of aggrecanase transcription (8), and its activity is regulated by IFT88, but only in response to cytokine (38). Recently, in chondrocytes, the JNK2 pathway has been linked to OA protease activity (9); however, we find that IFT88*^ORPK^* cells do not have constitutive JNK2 activity. Another possibility is the constitutive activation of the Hh pathway, recently linked to altered matrix catabolism and OA. Its activation results in spontaneous OA and pathway attenuation reduces the severity of surgically induced OA (35); however, this might not be a direct effect on aggrecanase activity as this group went on to demonstrate that Hh signaling regulates cholesterol accumulation in chondrocytes, associated with OA severity (53). IFT88 transduces Hh signal by ciliary trafficking of patched, smoothened, and the resultant modification of Gli transcription factors (54, 55). In our experimental conditions, the pathway is inhibited, likely owing to low-level Hh activation that is diminished by the loss of cilia; ∼85% of chondrocytes with this mutation do not assemble a primary cilium. In the absence of a likely post-transcriptional mechanisms.

Recent studies have linked increases in protease ativity in cartilage to the LRP-1 endocytosis system (11–13). More broadly, links between the cilium and endocytosis have also been described (14, 22, 24–27). In particular, Schou *et al.* (27) describes a caveolin-enriched microdomain at the ciliary transition zone that regulates Hh signal transduction within the ciliary axoneme. This endocytotic domain requires the novel ciliary kinesin-3 member kinesin family member 13B, which, of interest, also regulates LRP-1 (56); however, a functional link between ciliary machinery and the regulation of global cell endocytosis remains unreported. We propose that our findings link IFT88 and, by extension, potentially the primary cilium to increased matrix turnover that is similar to that observed in OA *via* an LRP-1 regulatory axis. Cilia are highly conserved in their structure throughout evolution; primary and motile cilia bear great similarities to prokaryotic and eukaryotic flagella. Trypanosomes are unicellular parasitic protozoa that assemble a flagellum for locomotion. Interestingly, the only site for endocytosis in trypanosomes is the flagellum pocket at the base of the flagellum structure, and on the deletion of IFT88, while a pocket is still assembled, endocytosis function is impaired (28). In chondrocytes, *in vitro*, we find that labeled ADAMTS-5 is taken up preferentially to 1 segment of the cell. Moreover, relative rates of endocytosis of both ADAMTS-5 and MMP-13 are reduced when IFT88 is disrupted, an effect that is comparable to that of LRP-1 inhibition by RAP. The experimental concentration of ligands is high compared with likely pathophysiologic levels. Nevertheless, an extension of protease half-life in cartilage that is comparable to that observed in these experiments is likely influential, increasing exposure time of the extracellular matrix to proteases. We also found increased LRP-1 shedding when IFT88 was disrupted. This, combined with reduced clearance, is likely to amplify the catabolic activity of the cells, as shed LRP-1α binds proteases and further increases their activity (12). IFT88*^ORPK^* chondrocytes express elevated levels of MMP-14, a sheddase that has been identified as disturbing the LRP-1 system in OA (12). Strikingly, we find the transmembrane-containing β chain of the LRP-1 receptor is concentrated in the region around the cilium. This distribution varies in different culture conditions but is pronounced in quiescent cells of the line used here and freshly isolated primary human chondrocytes. The IFT88*^ORPK^* mutation disrupts this LRP-1β organization without an effect on the amount of protein. We find that the surface population of this receptor displays this polarized distribution, often forming a hot spot for endocytotic clearance, rather than being purely reflective of a predictable relative concentration of all vesicular trafficking in the area around the MTOC. In cells that demonstrate highly concentrated LRP-1, we see staining that is supportive of a working hypothesis that LRP-1 lines the ciliary pocket region, but higher resolution microscopy will be required to determine if this is the case. Given the generalized associations between the cilium and endocytosis recognized by others, we do not interpret our results to be specific to LRP-1 or its ligands, but in the chondrocyte, these are particularly physiologically and disease relevant, hence our focus here. We cannot rule out a nonciliary mechanism. IFTs are linked to endocytosis independent of cilia. In T cells that do not form a cilium, but rather form an immune synapse responsible for the recycling of endosome-associated T-cell antigen receptors, IFT88 has been associated with polarized receptor recycling alongside IFT20, IFT57, and IFT52 (57–60). Ongoing studies are exploring the roles of other components of the ciliome in protease activity and endocytosis.

In summary, the current study makes a functional link between IFT and protease activity in chondrocytes *via* a regulatory axis that is highly relevant to osteoarthritis; however, this link between IFT and LRP-1–mediated endocytosis may be relevant in other pathophysiologic scenarios in which LRP-1 plays putative roles, such as in atherosclerotic and neuronal plaques (61). Additional studies are required to dissect this mechanism in detail, especially to identify a means by which to exploit this putative regulation of endocytosis by IFT.

## Abbreviations

ADAM: a disintegrin and metalloproteinase
ADAMTS: a disintegrin and metalloproteinase with thrombospondin motif
EEA-1: early endosome antigen 1
GLI2: glioma-associated oncogene family zinc finger 2
Hh: Hedgehog
IFT88: intraflagellar transport protein 88
LRP-1: LDL receptor–related protein 1
MMP: matrix metalloproteinase
MTOC: microtubule organizing center
OA: osteoarthritis
*ORPK*: Oak ridge polycystic kidney
PTCH1: patched-1
RAP: human receptor-associated protein
TIMP3: tissue inhibitor of metalloproteinase 3
